# Eradication of *P. aeruginosa* biofilm in endotracheal tubes based on lock therapy: results from an in vitro study

**DOI:** 10.1186/s12879-017-2856-0

**Published:** 2017-12-04

**Authors:** María Jesús Pérez-Granda, María Consuelo Latorre, Beatriz Alonso, Javier Hortal, Rafael Samaniego, Emilio Bouza, María Guembe

**Affiliations:** 1 Cardiac Surgery Postoperative Care Unit, Hospital General Universitario Gregorio Marañón, Madrid, Spain; 2 Instituto de Investigación Sanitaria Gregorio Marañón, Madrid, Spain; 3 CIBER Enfermedades Respiratorias-CIBERES (CB06/06/0058), Madrid, Spain; 4 Biology Department, School of Biology, Universidad Complutense de Madrid, Madrid, Spain; 5 Department of Clinical Microbiology and Infectious Diseases, Hospital General Universitario Gregorio Marañón, Madrid, Spain; 6 Confocal Laser Scanning Microscopy Unit, Hospital General Universitario Gregorio Marañón, Madrid, Spain; 7 Medicine Department, School of Medicine, Universidad Complutense de Madrid, Madrid, Spain; 8 Servicio de Microbiología Clínica y Enfermedades Infecciosas, Hospital General Universitario “Gregorio Marañón”, C/. Dr. Esquerdo, 46, 28007 Madrid, Spain

**Keywords:** *Pseudomonas aeruginosa* biofilm, Ventilator-associated pneumonia, Selective decontamination solution, Lock therapy, Confocal laser scanning microscopy

## Abstract

**Background:**

Despite the several strategies available for the management of biofilm-associated ventilator-associated pneumonia, data regarding the efficacy of applying antibiotics to the subglottic space (SS) are scarce. We created an in vitro model to assess the efficacy of antibiotic lock therapy (ALT) applied in the SS for eradication of *Pseudomonas aeruginosa* biofilm in endotracheal tubes (ETTs).

**Methods:**

We applied 2 h of ALT to a *P. aeruginosa* biofilm in ETTs using a single dose (SD) and a 5-day therapy model (5D). We used sterile saline lock therapy (SLT) as the positive control. We compared colony count and the percentage of live cells between both models.

**Results:**

The median (IQR) cfu counts/ml and percentage of live cells in the SD-ALT and SD-SLT groups were, respectively, 3.12 × 10^5^ (9.7 × 10^4^-0) vs. 8.16 × 10^7^ (7.0 × 10^7^-0) (*p* = 0.05) and 53.2% (50.9%-57.2%) vs. 91.5% (87.3%-93.9%) (*p* < 0.001). The median (IQR) cfu counts/ml and percentage of live cells in the 5D-ALT and 5D-SLT groups were, respectively, 0 (0-0) vs. 3.2 × 10^7^ (2.32 × 10^7^-0) (*p* = 0.03) and 40.6% (36.6%-60.0%) vs. 90.3% (84.8%-93.9%) (*p* < 0.001).

**Conclusion:**

We demonstrated a statistically significant decrease in the viability of *P. aeruginosa* biofilm after application of 5D-ALT in the SS. Future clinical studies to assess ALT in patients under mechanical ventilation are needed.

## Background

Ventilator-associated pneumonia (VAP) is one of the most common nosocomial infections, with significant morbidity and mortality. Between 9% and 27% of intubated patients develop VAP, although the incidence can increase to 46% in patients who require mechanical ventilation for more than 48 h after major heart surgery (MHS) [1, 2].

Bacterial biofilm is thought to be responsible for the development of respiratory infections in more than 80% of cases, with approximately 10^6^ bacterial cells/cm recovered from the endotracheal tube (ETT) [3]. In recent years, the association between VAP and biofilm has been well described, since pneumonia is associated more with the presence of biofilm than with duration of intubation [4, 5].

The various strategies for the prevention and treatment of VAP-associated biofilm include selective digestive decontamination (SDD), subglottic aspiration, antimicrobial drug–coated tubes, and devices that help to remove mucus, all of which can reduce biofilm formation in intubated patients [6–12]. Recent in vitro studies based on new designs of ETT have also described promising results regarding prevention of biofilm adhesion [13–16].

Despite the combination of compounds in the SDD solution would cover most microorganisms causing VAP and it even has not been associated to an increase of antibiotic resistance [17–20], it appears that SDD alone is not enough to reduce biofilm, as it only reaches the internal surface of the ETT [21, 22]. Therefore, an additional therapy with SDD solution directly applied at the external surface of the subglottic space can be a promising approach. Antibiotic lock therapy (ALT) is used for the prevention and treatment of catheter-related bacteremia [23–26], but data on the use of an SDD solution as ALT in the prevention and treatment of VAP are scarce [27].

Therefore, our objective was to apply an in vitro bench top model to assess the efficacy of SDD solution applied in the subglottic space for eradication of *Pseudomonas aeruginosa* biofilm in ETTs.

## Methods

Our prospective in vitro study was carried out in the laboratory of the Clinical Microbiology and Infectious Diseases Department, Hospital Gregorio Marañón, Madrid, Spain.

### Laboratory procedure

**Fig. 1:**
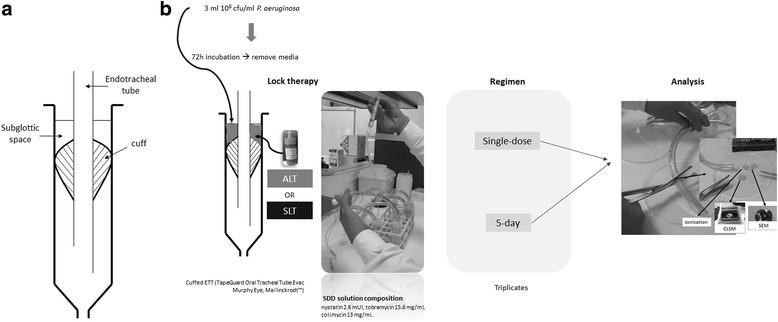
**a**. Schematic diagram of the bench top model simulating adult tracheal intubation based on a cuffed ETT (TaperGuard Oral Tracheal Tube Evac Murphy Eye, Mallinckrodt ™). **b**. Laboratory procedure. ALT with SDD or SLT were applied for 2 h during a single dose or a 5-day therapy at the subglottic space after the formation of a 72 h-mature biofilm of *P. aeruginosa.* After lock therapy, the subglottic area of the ETT was cut into 3 0.5 cm-segments for analysis

We used a bench top in vitro model simulating adult tracheal intubation based on a cuffed ETT (TaperGuard Oral Tracheal Tube Evac Murphy Eye, Mallinckrodt ™) (Fig. 1a) [7]. The ETT was colonized with 3 ml of a solution of 10^8^ cfu/ml of *Pseudomonas aeruginosa* ATCC 15442 in brain-heart infusion (BHI) and incubated at 37 °C for 72 h. BHI was discarded and replaced on each day of incubation. The model was run 4 times in order to test the following therapies: single dose of ALT (SD-ALT), 5 days of ALT (5D-ALT), single dose of sterile saline (SD-SLT), and 5-day SLT (5D-SLT). Sterile saline was used as a positive control (untreated). Each experiment was tested in triplicate (Fig. 1b).

Lock therapy consisted of a 2-h application of 3 ml of either SDD solution (nystatin 2.6 mIU, tobramycin 15.6 mg/ml, and colimycin 13 mg/ml) or sterile saline in the subglottic space. In the case of 5D-ALT and 5D-SLT, we used BHI between lock therapy periods. We also assessed whether SDD solution or sterile saline leakage occurred during lock therapy.

Once lock therapy had finished, the solution was removed, and the ETTs were washed with sterile saline. The subglottic area of the ETT was then cut into 3 segments, each measuring 0.5 cm (Fig. 1b). One segment was sonicated in order to analyze the colony counts (cfu/ml) by culture and the percentage of live cells in the sonicate by image. The remaining 2 segments were processed to visualize biofilm biomass and sessile cell structure using microscopy.

### Analysis of colony counts and live cells in the sonicate

Sonication was performed in 2 ml of buffer solution for 1 min at 50 Hz. For culture, the solution was serially diluted and cultured on blood agar plates, which were incubated for 24 h at 37 °C. Colony counts were expressed on a logarithmic scale as the number of cfu/ml.

For the analysis of the live/dead cells, the remaining sonicate was centrifuged (after culture), and the pellet was resuspended in 50 μl of sterile saline and stained using the Live/Dead® *Bac*Light kit™ (BacLight kit™; Invitrogen, Barcelona, Spain) for 15 min protected from the light. Staining was performed using 0.5 μl of SYTO® 9 (stock 3.34 mM DMSO) and 0.5 μl propidium iodide (stock 20 mM DMSO) in 20 μl of sample mounted on coverslips and imaged using confocal laser scanning microscopy (CLSM) in an inverted confocal fluorescence microscope (SPE, Leica Microsystems) equipped with ACS APO 10×/0.30 and ACS APO 63X/1.30 objectives. Samples were imaged using an ACS APO 63X/1.30 objective. During imaging, SYTO 9 emits green fluorescence and is used to identify living microorganisms with an intact membrane, whereas propidium iodide emits red fluorescence and stains dead bacteria with a damaged membrane. We analyzed 3 CLSM images of each sample. FIJI software (National Institute of Health, US) was used for image quantification. The percentage of live bacteria was calculated as the ratio between the number of live cells and the total number of cells ×100.

### Visualization of biofilm biomass

For visualization of the biofilm biomass in the ETT, we inactivated the ETT segments by freezing at −80 °C for 72 h. Then, after thawing for 30 min, the segments were stained with the Live/Dead® *Bac*Light kit™ for 15 min protected from the light. Staining was performed using 1.5 μl of SYTO® 9 (stock 3.34 mM DMSO) and 1.5 μl propidium iodide (stock 20 mM DMSO) in 1 ml phosphate-buffered saline. The samples were mounted on coverslips and imaged using CLSM at ACS APO 10X/0.30 objective [28, 29]. We displayed 6 CLSM images for each sample. Images were edited using FIJI software (National Institute of Health, US).

### Visualization of sessile cell structure

Biofilm structure was imaged using a 0.5-cm half section of the ETT segment fixed in 2.5% glutaraldehyde and then in osmium tetroxide (1%) and potassium ferricyanide (0.8%). The samples were then dehydrated in graded alcohol and sputter-coated with gold atoms (Taab Laboratories Equipment Ltd., Berks, UK). Samples were imaged via a scanning electron microscope (SEM, T300, Jeol Ltd., Tokyo, Japan). We displayed 6 SEM images for each sample.

### Statistical analysis

Qualitative variables appear with their frequency distribution*.* Quantitative variables are expressed as the median and interquartile range (IQR). Non-normally distributed continuous variables were compared using the Kruskal-Wallis and Mann-Whitney tests. The chi-square or Fisher exact test was used to compare categorical variables.

All statistical tests were 2-tailed. Statistical significance was set at *p* < 0.05 for all the tests. The statistical analysis was performed with SPSS 21.0.

## Results and discussion

### Single-dose lock therapy

**Fig. 2:**
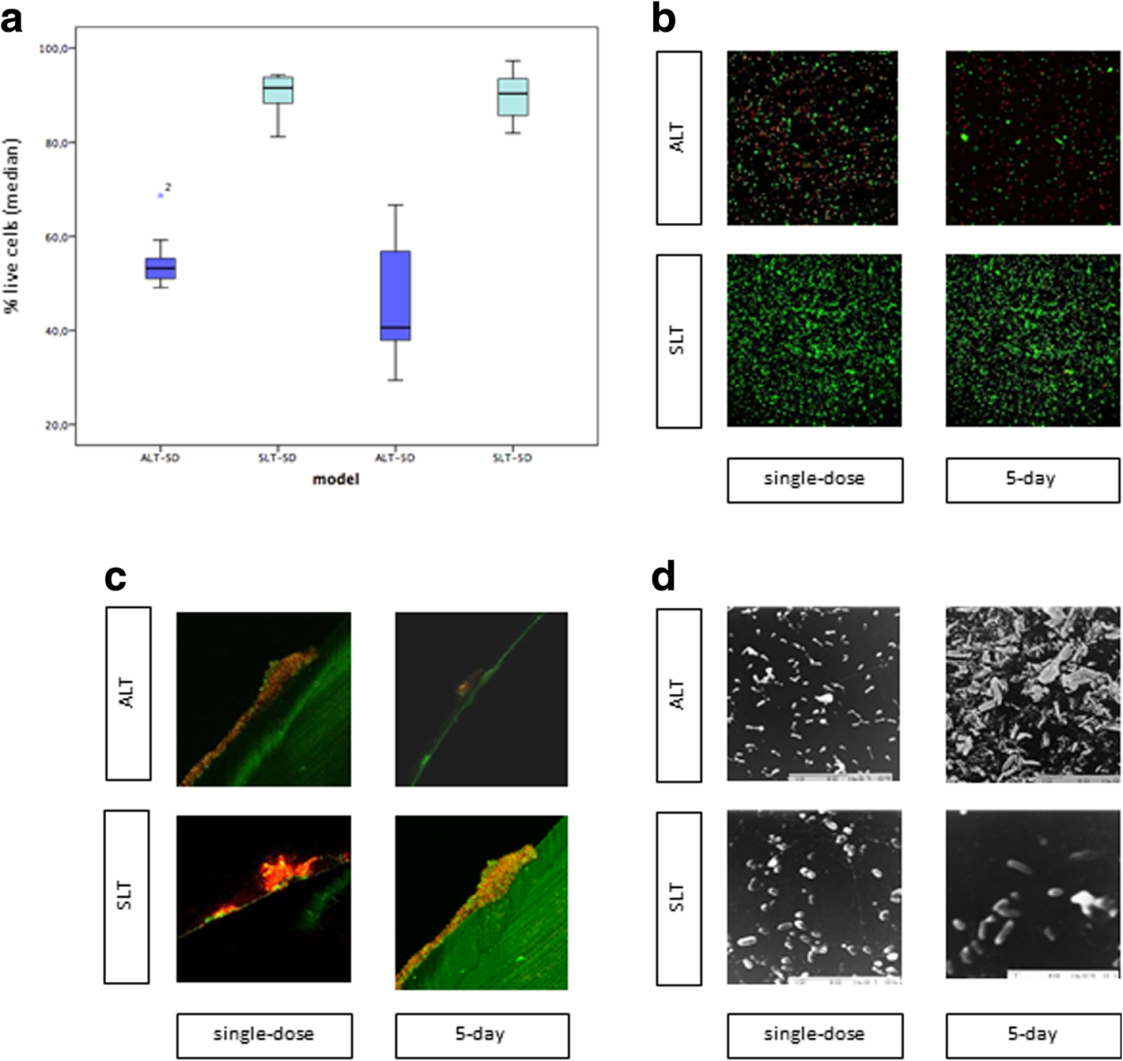
**a**. Comparison of percentage of live cells between the lock therapy models. *P* values were calculated by comparing the median (IQR) values using the Kruskal-Wallis test. **b**. Confocal laser scanning micrograph of treated and non-treated biofilms of *P. aeruginosa* recovered from the sonicate of the endotracheal tube segments. Samples of the sonicate were stained using the Live/dead® *Bac*Light kit™ (magnification ×1500). Bacteria recovered from 3 different ETTs were quantified (over 1000 cells per condition of single dose lock therapy and 5-day lock therapy). A single representative picture depicting the highest cell counts is reported for each model. **c**. Confocal laser scanning micrograph of the external surface of endotracheal tubes in the lock therapy and control groups. Samples were stained using the Live/dead® *Bac*Light kit™ (magnification ×250). The white arrow indicated the endotracheal tube wall. A single representative picture depicting the greatest biofilm accumulation is reported for each model. **d**. Scanning electron micrographs of the external surface of the endotracheal tubes in the lock therapy and control groups. Samples were fixed in glutaraldehyde, dehydrated in graded alcohol, and sputter-coated with gold atoms (magnification ×1500). The white arrow indicated the distortion of sessile cells. A single representative picture depicting the greatest cell deformation is reported for each model. **ALT**, antibiotic lock therapy; **SLT**, saline lock therapy; **SD**, single dose; **5D**, 5-day

In the SD lock therapy model, culture of the sonicate of the ETT segments yielded a median (IQR) of 2.15 × 10^5^ (9.7 × 10^4^-0.0) cfu/ml in the SD-ALT group and 8.16 × 10^7^ (7.0 × 10^7^-0.0) cfu/ml in the SD-SLT group (*p* = 0.05). The median (IQR) percentage of live cells detected by CLSM was 53.2% (50.9%-57.2%) in the SD-ALT group and 91.5% (87.3%-93.9%) in the SD-SLT group (*p* < 0.001) (Fig. 2a, b).

The samples of ETT segments observed under CLSM showed a similar biomass thickness in *P. aeruginosa* biofilm for both SD-ALT and SD-SLT, as also shown by SEM. This finding correlated with the median (IQR) number of total cells: SD-ALT, 915.0 (279.0-1074.5) vs. SD-SLT, 246.0 (168.5-1130.5) (*p* = 0.35) (Fig. 2c). However, the *P. aeruginosa* cells in the SD-ALT group were deformed compared with the cells in the control group (Fig. 2d).

### 5-day lock therapy

Five-day lock therapy yielded a median (IQR) cfu/ml from the sonicate of the ETT segments of 0.0 (0.0-0.0) in the 5D-ALT group and 3.2 × 10^7^ (2.32 × 10^7^-0.0) in the 5D-SLT group (*p* = 0.03). The median (IQR) percentage of live cells in each group was 40.6% (36.6%-60.0%) for 5D-ALT and 90.3% (84.8%-93.9%) for 5D-SLT (*p* < 0.001) (Fig. 2a, b).

We also found statistically significant differences in the median (IQR) number of total cells: SD-ALT, 32.0 (28.0-35.5) vs. SD-SLT, 50.0 (36.5-57.5) (*p* = 0.01). This finding can be observed in the CLSM images of the ETT segments, in which the thickness of the biomass was lower for 5D-ALT (Fig. 2c).

In the SEM images of Fig. 2d, the bacilli of *P. aeruginosa* were almost eradicated, and the sample was composed mainly of crystals derived from the used fluids.

### Comparison between single-dose and 5-day lock therapy

In order to assess whether 5D-ALT was better than SD-ALT, we compared only the median between the percentage of live cells and the number of cfu/ml, as SD and 5D therapies were not comparable according to the total number of cells in both the ALT group and the SLT group. We found that 5D-ALT was significantly better than SD-ALT in terms of the median (IQR) cfu/ml recovered in the sonicate: 0.0 (0.0-0.0) vs. 2.15 × 10^5^ (9.7 × 10^4^-0.0) (*p* = 0.04). Although there were no statistically significant differences in the median (IQR) percentage of live cells, 5D-ALT was slightly better than SD-ALT: 40.6% (36.6%-60.0%) vs. 53.2% (50.9%-57.2%) (*p* = 0.12).

When we analyzed whether leakage of lock solutions occurred through the ETT, we did not recover any other solution in either the SD or the 5D lock therapies.

Our in vitro data demonstrated that 5D-ALT using SDD solution applied on mature *P. aeruginosa* biofilm (72 h) in ETTs significantly reduced cell viability.

VAP is considered a major nosocomial infection, with a frequency ranging from 9% to 46% [2, 30, 31]. The highest frequencies are described among patients in MHS intensive care units (MHS-ICUs), as demonstrated in previous studies, where the risk of VAP increased among patients undergoing mechanical ventilation for more than 16.6 h after MHS [2, 32].

Biofilm formation on the ETT surface plays a key role in the pathogenesis of VAP, as it prevents the action of antibiotics and host defense cells [33]. Persistence of biofilm in the ETT of microorganisms potentially causing VAP is a common phenomenon, and, despite the use of systemic and inhaled antibiotics, patients have a worse clinical response [34].

Several preventive measures, such as subglottic aspiration and SDD, have proven effective in the reduction of VAP rates among patients under mechanical ventilation [35]. In addition, it was recently demonstrated that the use of SDD in intubated patients admitted to MHS-ICUs significantly reduced the frequency of VAP episodes [11]. However, data on the possible role of SDD solution as lock therapy in the subglottic space are scarce. A clinical study by Pneumatikos et al. demonstrated that continuous infusion of an antibiotic solution in the subglottic space significantly reduced VAP rates [27]. In our study, we used sonication and CLSM to demonstrate that a 2-h application of the SDD solution in ETT contaminated with a mature *P. aeruginosa* biofilm was associated with a considerable reduction in the number of viable cells (both cfu/ml and percentage of live cells). As for duration of lock therapy, even though the median percentage of live cells in the sonicate of the ETT segments treated with SD-ALT was significantly lower than in the control group (53.2% vs. 91.5%, *p* < 0.001), 5D-ALT not only significantly reduced the median percentage of live cells by >50% (40.6% vs. 90.3%, *p* < 0.001), but it also reduced the median number of cfu/ml (0.0 vs. 3.2 × 10^7^, *p* = 0.03). Our findings were also corroborated by direct observation of the biofilm in the ETT segments using CLSM and SEM, as 5D-ALT had a thinner biomass and the bacillus was deformed. This major cell damage observed by SEM was caused by tobramycin and colimycin, since both drugs produced leakage of cellular components, with the result that the cells were deformed [36].

We believe that the difference in the total number of cells observed in both periods of lock therapy is due to marked dislodgement of dead bacteria from the biofilm on the ETT surface in the 5D model [37]. Moreover, it was important to notice the difference between the cfu/ml obtained from cultures and the percentage of live cells of the sonicate. This can be a limitation of the study due to the possible presence of viable but no-culturable cells (VBNC) or due to a lack of use of a neutralizing fluid during sample extraction, but this last procedure could have affected also the biofilm structure by dislodgement [38].

Finally, even though our in vitro model was based on a static pre-established *P. aeruginosa* biofilm, our data support using the ALT not only as a therapeutic measure once the biofilm is established, but also as a preventive measure in patients who are expected to be under mechanical ventilation for more than 48 h (from the beginning of intubation until extubation or until discharge from the ICU). Moreover, the combination of compounds in the SDD solution would cover most microorganisms causing VAP. However, despite in ICUs with low levels of antibiotic resistance there is no evidence that universal use of SDD increases antibiotic resistance, in ICUs with high endemic levels of antibiotic resistance, SDD may increase the selective pressure for antibiotic-resistant microorganisms [17–20]. Routine prophylactic use of antibiotics should be carefully introduced in hospital settings where there are high levels of antibiotic resistance [1]. Besides, the in vitro efficacy of this therapy on the biofilm of other microorganisms causing VAP (eg, *Escherichia coli* and *Staphylococcus aureus*) should be tested.

## Conclusion

This is the first in vitro study demonstrating by culture and microscopy that *P. aeruginosa* biofilm in ETT can be significantly eradicated after applying ALT with SDD solution in the subglottic space. Future studies are needed to further evaluate the efficacy of this approach when combined with SDD as a prophylactic measure for VAP in patients under mechanical ventilation.

## Abbreviations

5D: 5-day
ALT: Antibiotic lock therapy
CLSM: Confocal laser scanning microscopy
ETT: Endotracheal tube
SD: Single dose
SDD: Selective digestive decontamination
SEM: Scanning electron microscopy
SLT: Saline lock therapy
